# The Role of Ultrasound as a Predictor of Malignancy in Indeterminate Thyroid Nodules—A Multicenter Study

**DOI:** 10.3390/medicina61061082

**Published:** 2025-06-12

**Authors:** Reem J. Al Argan, Dania M. Alkhafaji, Feras M. Almajid, Njoud K. Alkhaldi, Zahra A. Al Ghareeb, Moutaz F. Osman, Manal A. Hasan, Safi G. Alqatari, Abrar J. Alwaheed, Fatima E. Ismaeel, Reem S. AlSulaiman

**Affiliations:** 1 Department of Internal Medicine, College of Medicine, Imam Abdulrahman Bin Faisal University, King Fahad Hospital of the University, Dammam 31441, Eastern Province, Saudi Arabia; dmkhafaji@iau.edu.sa (D.M.A.); mahasan@iau.edu.sa (M.A.H.); sagqatari@iau.edu.sa (S.G.A.); ajwaheed@iau.edu.sa (A.J.A.); drfatimaebrahim@gmail.com (F.E.I.); rsolaiman615@gmail.com (R.S.A.); 2 Department of Surgery, College of Medicine, Imam Abdulrahman Bin Faisal University, King Fahad Hospital of the University, Dammam 31441, Eastern Province, Saudi Arabia; fmalmajid@iau.edu.sa; 3 Department of Internal Medicine, King Fahad Specialist Hospital-Dammam, Dammam 32253, Eastern Province, Saudi Arabia; dr.njoudalkhaldi@gmail.com; 4 Department of Internal Medicine, Qatif Central Hospital, AlQatif 32654, Eastern Province, Saudi Arabia; zaghareeb@gmail.com; 5 Department of Internal Medicine, King Fahad Military Medical Complex, Dhahran 34313, Eastern Province, Saudi Arabia; moutazosman@hotmail.com

**Keywords:** thyroid malignancy, indeterminate nodules, Bethesda III, Bethesda IV, ultrasound

## Abstract

*Background and Objectives:* Indeterminate thyroid nodules (Bethesda III and IV) are a common clinical entity that present a diagnostic challenge due to their intermediate risk of malignancy. This study aimed to evaluate the role of ultrasound in risk stratification and malignancy prediction to support clinical decision-making and reduce unnecessary surgical interventions. *Materials and Methods:* This retrospective multicenter cohort study included patients aged ≥18 years who underwent thyroid surgery between 2016 and 2022 at four centers in the Eastern Province of Saudi Arabia. Only nodules with indeterminate cytology (Bethesda III or IV) were included. Data collected included demographic characteristics, thyroid function, ultrasound features, cytology results, and histopathological findings. *Results:* A total of 679 patients with 733 nodules were reviewed. Of these, 206 patients with 223 indeterminate nodules were included (median age: 42 years; 88.3% female). The overall malignancy rate was 46.6%. Independent predictors of malignancy included solid hypoechoic composition (OR = 2.26, *p* = 0.012), microcalcifications (OR = 3.07, *p* = 0.002), lymph node involvement (OR = 2.43, *p* = 0.021), American Thyroid Association (ATA) intermediate to high suspicion category (OR = 1.9, *p* = 0.018), and Thyroid Imaging Reporting and Data Systems (TI-RADS) categories 4–5 (OR = 2.3, *p* = 0.003). Solid hypoechoic nodules showed 82.3% specificity and 63.0% positive predictive value (PPV); microcalcifications demonstrated 84.1% specificity and 68.4% PPV; lymph node involvement had 87.6% specificity and 68.9% PPV. The ATA and TI-RADS classifications showed higher sensitivity (63.5% and 68.0%, respectively), but lower specificity (53.1% and 52.8%, respectively). *Conclusions:* Ultrasound features, particularly solid hypoechoic composition, microcalcifications, and lymph node involvement, as well as ATA and TI-RADS classifications, were independent predictors of malignancy in indeterminate thyroid nodules. Although ATA and TI-RADS offered higher sensitivity, individual features demonstrated greater specificity and PPV. These findings support the use of ultrasound risk stratification to guide surgical decisions in high-risk cases and suggest that additional diagnostic evaluation may be appropriate for low-risk nodules.

## 1. Introduction

Thyroid nodules are commonly encountered in clinical practice. Studies have reported that palpable thyroid nodules occur in approximately 4–7% of the general population [1]. However, when more sensitive detection methods such as ultrasound are employed, the prevalence increases markedly, reaching 19–67% among randomly selected individuals [2,3]. The risk of malignancy in thyroid nodules varies considerably based on imaging characteristics and cytopathological findings [4,5,6,7]. According to the American Thyroid Association (ATA) classification for thyroid nodules, the malignancy risk ranges from less than 10% in low-suspicion nodules to over 70–90% in high-suspicion nodules based on ultrasound features [5]. The Bethesda System for Reporting Thyroid Cytopathology similarly stratifies malignancy risk, ranging from 0–3% for benign nodules (Bethesda II) to 97–99% for malignant nodules (Bethesda VI) [7].

Current guidelines use the Bethesda System for Reporting Thyroid Cytopathology to inform the management of thyroid nodules [5,6]. However, indeterminate thyroid nodules, categorized as Bethesda III [Atypia of Undetermined Significance or Follicular Lesion of Undetermined Significance] and Bethesda IV (Follicular Neoplasm or Suspicious for Follicular Neoplasm), have considerable diagnostic and management challenges because of their intermediate malignancy risk and the absence of definitive diagnostic tools. Studies have estimated the prevalence of indeterminate nodules to be between 10% and 25% [7,8,9]. The malignancy risk is reported to range from 6% to 18% for Bethesda III and from 10% to 40% for Bethesda IV, excluding noninvasive follicular thyroid neoplasm with papillary-like nuclear features, which has been reclassified as a nonmalignant entity in the revised Bethesda System [7]. The ATA recommends further evaluation of indeterminate nodules using a combination of clinical risk factors, ultrasound characteristics, repeat fine-needle aspiration (FNA), and molecular testing to refine malignancy risk estimates. When the risk remains substantial, diagnostic surgery (lobectomy or total thyroidectomy) may be warranted. Nodules assessed as low risk may instead be managed through active surveillance [5].

Ultrasound is a useful tool for predicting malignancy in thyroid nodules, particularly those with indeterminate cytology [10,11]. However, the supporting evidence remains inconsistent. In a study involving 187 patients with Bethesda III nodules, Alshahrani et al. identified several ultrasound features, including irregular margins, microcalcifications, multiple nodules, and hypoechogenicity, as crucial predictors of malignancy [12]. Similarly, Alyousif et al. reported that the Thyroid Imaging Reporting and Data System (TI-RADS) classification correlated significantly with outcomes of FNA and functioned as a reliable tool when molecular testing was unavailable [8]. By contrast, a retrospective study of 50 patients who underwent thyroid surgery for Bethesda III or IV nodules determined no statistically significant association between TI-RADS classification and malignancy risk [13].

These findings highlight the need for additional studies to clarify the role of ultrasound features in assessing malignancy risk in indeterminate thyroid nodules. The present study determined the predictive value of ultrasound in malignancy risk stratification to support more informed clinical decision-making, reduce the need for unnecessary diagnostic surgeries, and improve patient outcomes.

## 2. Materials and Methods

This retrospective multicenter cohort study included patients with thyroid nodules who were followed in endocrine clinics and underwent either total thyroidectomy or lobectomy between 1 January 2016 and 31 December 2022, at four centers in the Eastern Province of Saudi Arabia: King Fahad Hospital of the University (KFHU), King Fahad Specialist Hospital-Dammam (KFSH-D), Qatif Central Hospital (QCH), and King Fahad Military Medical Complex (KFMMC).

This study included patients aged ≥ 18 years who underwent thyroidectomy or lobectomy between 2016 and 2022 and had indeterminate thyroid nodules classified as Bethesda III or IV based on fine-needle aspiration cytology.

We excluded patients aged <18 years, pregnant patients, cases outside the study period, patients whose Bethesda classification was not consistent with class III or IV, and patients whose final pathology revealed incidental malignancy unrelated to the nodule identified on thyroid ultrasound.

### 2.1. Data Collection Procedure

After obtaining ethical approval from the respective committees at the participating centers, we collected data from electronic medical records available in each institution’s healthcare information system. The dataset included the following:

1. Baseline data (age and sex).
2. Thyroid hormonal status (hyperthyroidism, hypothyroidism, or euthyroid).
3. Thyroid function tests: thyroid-stimulating hormone (TSH), free thyroxine (T4), and free triiodothyronine (T3).
4. Preoperative ultrasound features of thyroid nodules. The US images were performed by US technicians as per hospital protocols and reported by US-specialized radiologists. Thereafter, during data collection, ultrasound images and reports were reviewed by an endocrinologist certified by the American Association of Clinical Endocrinologists (AACE) through the Endocrine Certification in Neck Ultrasound (ECNU). Based on these findings, nodules were stratified in accordance with the 2015 thyroid ATA classification system into high, intermediate, low, very low suspicion, and benign categories [5]. Nodules were also classified using the Thyroid Imaging Reporting and Data System (TI-RADS) Classification from class 1 to 5 [4].
5. Preoperative cytology results based on the Bethesda classification of thyroid FNA cytology [14].
6. Postoperative pathology reports, categorized as benign, premalignant, or malignant. In differentiated thyroid cancer cases, histopathological findings were further classified according to the 2015 ATA risk stratification for thyroid cancer recurrence [5].

Subsequently, we evaluated the role of ultrasound as a predictor of malignancy in indeterminate thyroid nodules by analyzing individual suspicious features and applying both the ATA and TI-RADS classification systems. Diagnostic performance was assessed by calculating sensitivity, specificity, positive predictive value (PPV), and negative predictive value (NPV).

### 2.2. Statistical Analysis

Data were analyzed using IBM Statistical Package for the Social Sciences (SPSS) version 22. The Kolmogorov–Smirnov test was used to assess the normality of the data. Categorical variables were reported as frequencies and percentages, while continuous variables were presented as medians with first (Q1) and third (Q3) quartiles. The chi-square test or Fisher’s exact test was used to evaluate associations between categorical variables, and the Kruskal–Wallis test was applied to compare medians. Odds ratios (ORs) with 95% confidence intervals (CIs) were calculated through multivariate regression analysis. A *p* value of less than 0.05 was considered statistically significant.

## 3. Results

### 3.1. Population Demographics and Underlying Thyroid Hormonal Status

A total of 679 patients with 733 thyroid nodules were initially identified. Of these, 206 patients with 223 nodules met the inclusion criteria and were included in the study. The median patient age was 42 years, and the majority were female (88.3%). Most patients were euthyroid (93.6%), while 4.9% were hypothyroid and 1.5% were hyperthyroid. Regarding nodule size, 76.7% measured less than 4.0 cm, whereas 23.3% were 4.0 cm or larger. Demographic characteristics and thyroid function status were comparable between Bethesda III and IV categories, with the exception of free T4 and free T3 levels, which were significantly higher in Bethesda IV nodules compared to Bethesda III (*p* = 0.001) (Table 1).

### 3.2. Ultrasound Features of Indeterminate Thyroid Nodules

Analysis of ultrasound features in indeterminate thyroid nodules showed that 65.9% were single, whereas 34.1% were multiple. Single nodules were more frequently observed in Bethesda III (74.6%) compared to Bethesda IV (53.8%, *p* = 0.001), whereas multiple nodules were more common in Bethesda IV (61.4%, *p* = 0.02).

Regarding nodule composition, the majority were solid (59.6%), followed by mixed composition (24.2%). Solid nodules were more prevalent in Bethesda IV (68.8%) than in Bethesda III (53.1%, *p* = 0.018). In contrast, mixed composition was more frequently observed in Bethesda III (29.2%) than in Bethesda IV (17.2%, *p* = 0.039). Extra-thyroid extension was significantly more common in Bethesda III (9.2%) and was absent in Bethesda IV (*p* = 0.003).

According to the ATA classification, 37.2% of nodules were categorized as intermediate or low suspicion. Intermediate-suspicion nodules were more frequent in Bethesda IV (52.7%) than in Bethesda III (26.2%, *p* = 0.001), whereas low-risk (43.1%, *p* = 0.03) and very low-risk nodules (10.8%, *p* = 0.01) were more common in Bethesda III category.

Based on the TI-RADS, most indeterminate nodules were classified as TI-RADS 4 (43.5%), followed by TI-RADS 3 (29.6%). Bethesda III nodules exhibited a significantly higher frequency of TI-RADS 1 (5.4%, *p* = 0.02) (Table 2).

### 3.3. Risk of Malignancy and Aggressiveness of Thyroid Cancer in Indeterminate Thyroid Nodules

Malignancy was identified in 104 of 223 indeterminate thyroid nodules, representing an overall malignancy rate of 46.6%. Bethesda IV nodules demonstrated a higher malignancy rate (53.8%) compared to Bethesda III nodules (41.5%), although this difference did not reach statistical significance (*p* = 0.07). Benign nodules were more frequently observed in Bethesda III (56.9%) than in Bethesda IV (41.9%, *p* = 0.02).

Papillary thyroid carcinoma (PTC) was the most common malignancy, accounting for 93.3% of malignant cases, followed by follicular thyroid carcinoma (FTC) in 5.8%. Of note, Bethesda IV nodules resulted in a higher percentage of FTC (10.0%) than Bethesda III nodules (1.9%), although it did not reach statistical significance (*p* = 0.08). Additionally, most PTC cases were classic variant (50.5%) followed by follicular variant in 43.3%.

Among high-risk histopathological features, 20.6% of malignant nodules were multifocal, 1.3% exhibited extra-thyroid extension, and 4.5% demonstrated lympho-vascular invasion. No significant differences in these features were found between Bethesda III and IV nodules.

According to the ATA classification for risk of recurrence of differentiated thyroid carcinoma, most indeterminate nodules were classified as low-risk (69.9%), while 26.8% were intermediate-risk and 5.2% were high-risk (Table 3).

### 3.4. Predictors of Malignancy in Indeterminate Thyroid Nodules (Univariate Analysis)

Univariate analysis revealed that malignant nodules were more likely to exhibit a solid composition (65.4% vs. 54.0%) and hypoechogenicity (46.2% vs. 33.6%), although these differences were not statistically significant. However, the combination of solid and hypoechoic features was significantly associated with malignancy (32.7% vs. 17.7%, *p* = 0.01). In contrast, mixed composition was significantly more common among benign nodules (31.9%, *p* = 0.012).

Among high-risk ultrasound features, microcalcifications were significantly associated with malignancy (37.5% vs. 15.9%, *p* = 0.003). A taller-than-wide shape (10.6% vs. 2.7%, *p* = 0.018) and lymph node involvement (29.8% vs. 12.4%, *p* = 0.002) were also strong predictors of malignancy.

Based on the ATA classification, malignant nodules were significantly associated with higher-risk ultrasound categories (intermediate to high suspicion) (63.5%, *p* = 0.017), whereas nodules categorized as very low to low suspicion were more frequently benign in pathology (52.7%, *p* = 0.02). Similarly, TI-RADS categories 4–5 were strongly associated with malignancy (68.0%, *p* = 0.002), while TI-RADS 2–3 nodules were more often benign (52.8% vs. 32.0%, *p* = 0.0026). These findings indicate the predictive value of ultrasound features in assessing the risk of malignancy (Table 4).

### 3.5. Predictors of Malignancy in Indeterminate Thyroid Nodules (Multivariate Analysis)

Multivariate analysis indicated that hypoechoic nodules were more likely to be malignant, with an odds ratio (OR) of 1.69; however, this association did not reach statistical significance (*p* = 0.06). In contrast, hyperechoic nodules were less likely to be malignant (OR 0.55, *p* = 0.059), suggesting a possible protective trend. Isoechoic nodules showed no significant association with malignancy (OR 0.66, *p* = 0.228). The combination of solid and hypoechoic features emerged as an independent predictor of malignancy (OR 2.26, *p* = 0.012). Additionally, the presence of microcalcifications (OR 3.07, *p* = 0.002) and lymph node involvement (OR 2.43, *p* = 0.021) were identified as strong independent predictors of malignancy.

Analysis of ultrasound classification systems further demonstrated that nodules categorized as intermediate to high suspicion under the ATA classification (OR 1.9, *p* = 0.018) and those classified as TI-RADS 4–5 (OR 2.3, *p* = 0.003) were also independent predictors of malignancy (Table 5).

### 3.6. Diagnostic Performance of Ultrasound Features and Risk Stratification Systems to Predict Malignancy

Various ultrasound features and risk stratification systems were evaluated for their diagnostic performance in predicting thyroid malignancy. Solid and hypoechoic nodules demonstrated a sensitivity of 32.7%, specificity of 82.3%, PPV of 63.0%, and NPV of 57.1%. Microcalcifications yielded a sensitivity of 37.5%, specificity of 84.1%, PPV of 68.4%, and NPV of 59.4%. The presence of suspicious lymph nodes was associated with a sensitivity of 29.8%, specificity of 87.6%, PPV of 68.9%, and NPV of 57.6%. For nodules categorized as intermediate to high suspicion under the ATA classification, sensitivity was 63.5%, specificity 53.1%, PPV 55.5%, and NPV 61.2%. The TI-RADS 4–5 category demonstrated the highest sensitivity among all parameters evaluated (68.0%), with a specificity of 52.8%, PPV of 57.6%, and NPV of 63.6% (Table 6).

## 4. Discussion

This retrospective multicenter cohort study included 206 patients with 233 indeterminate thyroid nodules and evaluated the role of ultrasound in predicting malignancy. In this cohort, demographic characteristics, gender distribution, thyroid function status, and nodule size were comparable between Bethesda III and IV nodules. However, Bethesda IV nodules demonstrated significantly higher levels of free T4 (median: 11.01 vs. 2.9) and free T3 (median: 3.8 vs. 3.0) (*p* = 0.001 for both). These hormonal elevations may reflect increased metabolic activity or early autonomous behavior in Bethesda IV nodules and suggest a potential role for thyroid hormone levels as adjunctive markers of malignancy risk when interpreted alongside ultrasound features. Our findings did not demonstrate a significant association between TSH levels and the risk of malignancy. In contrast, previous studies have suggested a positive correlation between elevated TSH levels and malignant thyroid nodules [15,16]. For example, Alarifi et al. reported that malignant nodules had significantly higher TSH levels compared to benign ones (1.94 vs. 1.62, *p* = 0.002). Additionally, they found that the likelihood of a thyroid nodule being malignant was 1.54 times greater in the presence of elevated TSH levels (*p* = 0.038) [15]. However, no prior studies have specifically investigated the association between thyroid hormone profiles and malignancy risk in indeterminate nodules, indicating the need for further research to explore this relationship.

Our study identified significant differences in ultrasound risk stratification between Bethesda III and IV nodules based on ATA and TI-RADS classifications. Intermediate-suspicion nodules were more frequently observed in Bethesda IV (52.7% vs. 26.2%, *p* = 0.001), whereas low-suspicion (43.1%, *p* = 0.03) and very low-suspicion nodules (10.8%, *p* = 0.01) were more prevalent in Bethesda III. TI-RADS 4 was the most common category overall, although Bethesda III nodules had a higher proportion of TI-RADS 1 nodules (5.4%, *p* = 0.02). Few studies have directly compared these features. For example, Alqahtani et al. reported that among benign nodules, the majority of Bethesda III nodules were classified as TI-RADS 3 (78.6%), while most Bethesda IV nodules were TI-RADS 4 (63.6%, *p* = 0.039), although no significant differences were observed among malignant nodules [13].

In our study, the overall malignancy risk among indeterminate thyroid nodules was 46.6%. Benign nodules were significantly more common in Bethesda III compared with Bethesda IV (56.9% vs. 41.9%, *p* = 0.02), resulting in a higher malignancy rate for Bethesda IV nodules (53.8%) than for Bethesda III nodules (41.5%). However, this difference did not reach statistical significance (*p* = 0.07). These findings are consistent with previous studies reporting malignancy rates for indeterminate nodules ranging from 27% to 53.3% [12,17,18,19,20]. One study of 221 patients with Bethesda IV nodules found a malignancy rate of 48.9% [20]. Additionally, a retrospective analysis of 217 patients who underwent ThyroSeq v3 molecular testing followed by thyroid surgery reported higher malignancy rates in Bethesda IV nodules (83.7%) compared to Bethesda III nodules (67.5%) [21]. Overall, our results align with the existing literature and support the conclusion that Bethesda IV nodules carry a greater risk of malignancy than Bethesda III nodules [22].

PTC was the most common malignancy in our cohort, accounting for 93.3% of all malignant indeterminate thyroid nodules, followed by other subtypes of thyroid cancer. Based on the ATA risk classification for thyroid cancer recurrence, 69.9% of malignant cases were categorized as low risk. These findings are consistent with those reported by Payne et al., who examined both Bethesda III and IV nodules and found that PTC constituted 69.6% and 75% of malignant cases in each category, respectively [21]. In that study, the majority of malignant nodules did not exhibit aggressive features, and no significant difference was observed between the two Bethesda groups (*p* = 0.573) [21].

We further examined several ultrasound features in relation to malignancy. Malignant nodules were more likely to be solid (65.4% vs. 54.0%) and hypoechoic (46.2% vs. 33.6%), although these associations were not statistically significant when evaluated individually. However, the presence of both solid and hypoechoic features within the same nodule was strongly associated with malignancy (32.7%, *p* = 0.01). In contrast, mixed-composition nodules were significantly more common in benign cases (31.9%, *p* = 0.012). Multivariate analysis confirmed that the combination of solid and hypoechoic features served as an independent predictor of malignancy (OR 2.26, *p* = 0.012). Alshahrani et al. similarly reported an association between hypoechogenicity and malignancy (37.9%, *p* = 0.044), although the difference in the prevalence of solid composition between malignant and benign nodules in their study (93.1% vs. 85%) did not reach statistical significance (*p* = 0.08) [12]. A separate retrospective study of 221 Bethesda IV cases identified hypoechogenicity (OR 1.64, *p* = 0.041) and solid composition (OR 8.4, *p* = 0.004) as significant risk factors for malignancy [20]. Our findings also demonstrated that the combined presence of solid and hypoechoic features yielded a sensitivity of 32.7%, specificity of 82.3%, PPV of 63.0%, and NPV of 57.1%. While previous studies have often evaluated these features independently, our findings were consistent with earlier reports with regard to specificity and PPV. For example, one study reported that marked hypoechogenicity alone had a sensitivity of 41.4% and a specificity of 92.2% [23], which aligns closely with our specificity value despite slight differences in sensitivity. These results indicate that solid and hypoechoic features are highly specific and useful for confirming malignancy; however, their limited sensitivity suggests they are not sufficient for excluding malignancy on their own. Therefore, the presence of these features should prompt further diagnostic evaluation to support clinical decision-making.

Our analysis showed that microcalcifications were significantly associated with malignancy in both univariate (37.5%, *p* = 0.003) and multivariate analyses (OR = 3.07, *p* = 0.002). Microcalcifications demonstrated a sensitivity of 37.5%, specificity of 84.1%, PPV of 68.4%, and NPV of 59.4%. These findings are consistent with prior evidence supporting an association between microcalcifications and malignancy, particularly in indeterminate nodules [12,22]. Alshahrani et al. reported the presence of microcalcifications in 59.8% of malignant indeterminate thyroid nodules, a statistically significant association (*p* < 0.001) [12]. Similarly, Remonti et al. identified microcalcifications and irregular margins as key predictors of malignancy, and within the subset of indeterminate nodules, both microcalcifications and central vascularity were predictive of malignancy, with a sensitivity of 39.5% and specificity of 87.8% [10]. Another study also reported that microcalcifications had higher specificity (75.9%) than sensitivity (44.1%) [11]. Collectively, these findings reinforce the diagnostic value of microcalcifications as a specific ultrasound feature that, although not highly sensitive, provides strong evidence for malignancy when present—particularly in nodules with indeterminate cytology.

A taller-than-wide shape, defined as an anteroposterior-to-transverse ratio ≥1 [24], was associated with malignancy in univariate analysis (10.6% vs. 2.7%, *p* = 0.018); however, this association was not sustained in multivariate analysis (OR = 2.81, *p* = 0.144). Previous studies have shown that this ultrasound feature is highly specific for malignancy in thyroid nodules, with reported specificity ranging from 91.4% to 96.6% [10,23], although sensitivity has varied between 40% and 68% [23,24]. A high negative predictive value, reaching up to 87.7%, has also been reported [24]. A retrospective single-center study involving 254 nodules demonstrated a strong independent association between a taller-than-wide shape and malignancy (OR = 25.3, *p* < 0.001) [25]. By contrast, Al Dawish et al. assessed this feature in Bethesda III nodules and found a 50% malignancy rate among nodules exhibiting a taller-than-wide shape; however, the association was not statistically significant (*p* = 0.476) [18].

Lymph node involvement was identified as a significant predictor of malignancy in both univariate (29.8%, *p* = 0.002) and multivariate analyses (OR = 2.43, *p* = 0.021). In our cohort, the presence of enlarged lymph nodes demonstrated a sensitivity of 29.8%, specificity of 87.6%, PPV of 68.9%, and NPV of 57.6%. These findings align with previous reports, including one study that identified enlarged cervical lymph nodes (>1 cm) in 46.5% of patients with PTC, compared to only 17.8% in those with benign nodules (*p* < 0.001) [26]. Other studies have reported even higher rates of lymph node enlargement in malignant nodules, reaching up to 72.7% [27]. The diagnostic performance observed in our study is comparable to earlier findings, which showed that the presence of enlarged cervical lymph nodes with at least one suspicious feature yielded a sensitivity of 33.33%, specificity of 83.02%, PPV of 70.97%, and NPV of 50.0% [26]. These results suggest that although lymph node enlargement is not a highly sensitive indicator, it is a highly specific one. Its presence should therefore raise strong clinical suspicion for malignancy and prompt further diagnostic evaluation.

Our analysis identified ATA intermediate-to-high suspicion categories (63.5%, *p* = 0.017; OR = 1.9, *p* = 0.018) and TI-RADS 4–5 classifications (68.0%, *p* = 0.002; OR = 2.3, *p* = 0.003) as independent predictors of malignancy in indeterminate thyroid nodules. These findings are consistent with previous studies that have demonstrated an association between ultrasound-based risk stratification systems and malignancy risk in both indeterminate nodules [8,18,28] and thyroid nodules more broadly [29]. For instance, one study evaluating Bethesda III and IV nodules reported significantly increased malignancy risk with higher TI-RADS scores, identifying TI-RADS 4 (OR = 2.59, *p* = 0.000) and TI-RADS 5 (OR = 29.03, *p* = 0.002) as independent predictors of malignancy [8]. However, not all studies have supported this association. A retrospective analysis of 110 Bethesda III nodules found no significant correlation between TI-RADS classification and final histopathological outcomes [30].

In our cohort, the ATA intermediate- to high-suspicion category demonstrated a sensitivity of 63.5%, specificity of 53.1%, PPV of 55.5%, and NPV of 61.2%. The TI-RADS 4–5 categories exhibited slightly higher sensitivity (68.0%) with comparable specificity (52.8%), PPV of 57.6%, and NPV of 63.6%. These findings are consistent with previous studies in terms of specificity but indicate somewhat lower sensitivity in our population. For comparison, prior studies have reported TI-RADS 4–5 sensitivity ranging from 77.9% to 91.7%, specificity between 52.8% and 57.3%, PPVs around 73.8%, and NPVs of 62.8% for predicting malignancy [29,31].

These results suggest that while individual sonographic features, such as solid and hypoechoic composition, microcalcifications, and lymph node involvement, demonstrate higher specificity, standardized risk stratification systems such as ATA and TI-RADS offer a more balanced diagnostic performance, particularly in terms of sensitivity. Additionally, our findings indicate that the ATA intermediate- to high-suspicion and TI-RADS 4–5 categories, despite having only moderate sensitivity and specificity, show a strong association with malignancy in multivariate analysis (OR = 1.9, *p* = 0.018, and OR = 2.3, *p* = 0.003, respectively), reinforcing their clinical relevance. The relatively high odds ratios suggest that nodules classified as high risk under these systems carry a substantially increased likelihood of malignancy. Although the negative predictive values (61.2% for ATA and 63.6% for TI-RADS) indicate that a portion of low-risk nodules may still be malignant, the strength of the association observed in high-risk categories supports their use in clinical decision-making. As such, nodules categorized as low risk should undergo careful follow-up and may warrant additional diagnostic evaluation, such as molecular testing, due to the moderate NPV. Conversely, nodules classified as high risk in either system should be considered for more aggressive management, given their strong independent association with malignancy.

Molecular testing is increasingly recommended in clinical guidelines [5,6,32] as it may help avoid unnecessary diagnostic surgery or identify patients with a high risk of malignancy who would benefit from surgical treatment. The latest European Thyroid Association (ETA) guidelines advise the use of molecular tests—when available—prior to surgery for indeterminate thyroid nodules, to support surgical decision-making [32]. Current molecular tests assess somatic mutations, gene expression profiles, and microRNA classifiers [32]. Notably, the use of molecular tests reduces unnecessary thyroid surgeries, with avoidance rates ranging from 50.3% to 68.6% [33].

## 5. Conclusions

Our study demonstrates that indeterminate thyroid nodules carry a substantial risk of malignancy, with Bethesda IV nodules exhibiting a higher malignancy rate than Bethesda III nodules. Several ultrasound features and risk stratification systems were found to be useful in predicting malignancy in this subgroup. Independent predictors included solid and hypoechoic composition, microcalcifications, and lymph node involvement, all of which demonstrated high specificity and PPV.

Furthermore, ATA intermediate- to high-suspicion and TI-RADS 4–5 categories were significantly associated with malignancy. While these classifications exhibited higher sensitivity compared to individual suspicious ultrasound features, they had comparatively lower specificity. Based on these findings, nodules with high-risk ultrasound features may warrant surgical referral. In contrast, nodules lacking such features may be better managed with additional risk assessment through molecular testing or close follow-up. This stratified approach may support more informed clinical decision-making and reduce the incidence of unnecessary surgeries.

## 6. Strengths and Limitations

This retrospective multicenter study provides meaningful insights, particularly due to its real-world, multicenter design. This enhances the generalizability of the findings and reflects contemporary clinical practice across diverse healthcare settings. The inclusion of multiple centers helps reduce single-center bias and supports the broader applicability of the results. Moreover, the study focuses on a clinically relevant population—patients with indeterminate thyroid nodules—and highlights the potential utility of ultrasound as a noninvasive, widely accessible diagnostic tool.

Several limitations should be acknowledged. The relatively small sample size may limit statistical power, and the retrospective design introduces risks of selection and information bias. In addition, due to the unavailability of molecular or genetic tests in the participating centers, the study did not incorporate these approaches, which are increasingly valuable in the risk stratification and management of indeterminate thyroid nodules. Future large-scale prospective studies are needed to provide stronger evidence on the clinical utility of ultrasound in this context.

## Figures and Tables

**Table 1:** Population demographics and underlying thyroid diseases.

		Total Frequency (Percentage)	Bethesda III Frequency (Percentage)	Bethesda IV Frequency (Percentage)	*p* Value
Age (years) Median (Q1–Q3) 42 (35–52)	18–40	88 (42.7%)	48 (41.4%)	40 (44.4%)	0.824
	41–60	92 (44.7%)	54 (46.6%)	38 (42.2%)	
	61–80	26 (12.6%)	14 (12.1%)	12 (13.3%)	
Sex	Male	24 (11.7%)	14 (12.1%)	10 (11.1%)	0.83
	Female	182 (88.3%)	102 (87.9%)	80 (88.9%)	
Underlying thyroid hormonal status	Hyperthyroidism	3 (1.5%)	2 (1.7%)	1 (1.2%)	0.7
	Hypothyroidism	10 (4.9%)	6 (4.96%)	4 (4.7%)	0.93
	Euthyroid	193 (93.6%)	113 (93.4%)	80 (94.1%)	0.83
Size of thyroid nodules	<4.0 cm	171 (76.7%)	104 (80%)	67 (72.04%)	0.166
	≥4.0 cm	52 (23.3%)	26 (20%)	26 (27.96%)	
Thyroid function test Median (Q1–Q3)	TSH (uIU/mL)	1.3 (0.9–2.7)	1.2 (0.85–2.3)	2 (0.88–3)	0.076
	Free T4 (ng/dL)	9.7 (1.2–12.5)	2.9 (1.06–12)	11.01 (2.8–13)	0.001
	Free T3 (pg/mL)	3.3 (2.5–4.2)	3.0 (2–3.74)	3.8 (2.8–4.4)	0.001

Abbreviations: Q1: first quartile; Q3: third quartile; TSH: thyroid-stimulating hormone; T4: thyroxine; T3: triiodothyronine.

**Table 2:** Ultrasound features of indeterminate thyroid nodules.

	Total Frequency (Percentage)	Bethesda III Frequency (Percentage)	Bethesda IV Frequency (Percentage)	*p* Value
Ultrasound features				
Number of nodules				
Single nodule	147 (65.9%)	97 (74.6%)	50 (53.8%)	0.001
Multiple nodules	76 (34.1%)	33 (42.3%)	43 (61.4%)	0.02
Nodule type				
Solid	133 (59.6%)	69 (53.1%)	64 (68.8%)	0.018
Cystic	36 (16.1%)	25 (19.2%)	11 (11.8%)	0.138
Mixed	54 (24.2%)	38 (29.2%)	16 (17.2%)	0.039
Nodule echogenicity				
Hypoechoic	89 (39.9%)	54 (41.5%)	35 (37.6%)	0.5
Hyperechoic	80 (35.9%)	43 (33.1%)	37 (39.8%)	0.45
Isoechoic	54 (24.2%)	33 (25.4%)	21 (22.6%)	0.65
Other high-risk features				
Solid and hypoechoic	57 (25.6%)	36 (27.7%)	21 (22.6%)	0.388
Microcalcifications	57 (25.6%)	38 (29.2%)	19 (20.4%)	0.12
Taller than wide	14 (6.3%)	7 (5.4%)	7 (7.5%)	0.52
Irregular margins	3 (1.3%)	3 (2.3%)	0 (0%)	0.51
Extra-thyroid extension	12 (5.4%)	12 (9.2%)	0 (0%)	0.003
Lymph nodes	47 (21.1%)	30 (23.1%)	17 (18.3%)	0.36
Ultrasound risk classification systems				
ATA risk classification				
Benign	1 (0.4%)	0 (0%)	1 (1.1%)	0.65
Very low suspicion	16 (7.2%)	14 (10.8%)	2 (2.2%)	0.01
Low suspicion	83 (37.2%)	56 (43.1%)	27 (29.0%)	0.03
Intermediate suspicion	83 (37.2%)	34 (26.2%)	49 (52.7%)	0.001
High suspicion	40 (17.9%)	26 (20.0%)	14 (15.1%)	0.28
TI-RADS classification				
TI-RADS 1	4 (1.8%)	7 (5.4%)	0 (0%)	0.02
TI-RADS 2	23 (10.3%)	2 (1.5%)	2 (2.2%)	0.69
TI-RADS 3	66 (29.6%)	11 (8.5%)	12 (12.9%)	0.28
TI-RADS 4	97 (43.5%)	42 (32.3%)	24 (25.8%)	0.29
TI-RADS 5	26 (11.7%)	52 (40%)	45 (48.4%)	0.23

Abbreviations: ATA: American Thyroid Association; TI-RADS: Thyroid Imaging Reporting and Data Systems.

**Table 3:** Risk of malignancy and aggressiveness of thyroid cancer in indeterminate thyroid nodules.

	Total Frequency (Percentage)	Bethesda III Frequency (Percentage)	Bethesda IV Frequency (Percentage)	*p* Value
Risk of malignancy				
Malignant	104 (46.6%)	54 (41.5%)	50 (53.8%)	0.07
Benign	113 (50.7%)	74 (56.9%)	39 (41.9%)	0.02
Pre-malignant (NIFTP)	6 (2.7%)	2 (1.5%)	4 (4.3%)	0.2
Postoperative pathology				
Type of thyroid cancer				
PTC	97 (93.3%)	52 (96.3%)	45 (90.0%)	0.2
FTC	6 (5.8%)	1 (1.9%)	5 (10.0%)	0.08
MTC	1 (0.9%)	1 (1.9%)	0 (0%)	0.32
PTC variants				
Classic	49 (50.5%)	28 (53.8%)	21 (46.7%)	0.48
Follicular	42 (43.3%)	20 (38.5%)	22 (48.9%)	0.3
Tall cell	2 (2.1%)	1 (1.9%)	1 (2.2%)	0.92
Others	4 (4.1%)	* 3 (5.8%)	^†^ 1 (2.2%)	0.37
Other high-risk features				
High-risk variant	6 (2.7%)	4 (3.1%)	2 (2.2%)	0.7
Unifocal	57 (25.1%)	31 (23.8%)	26 (28.0%)	0.41
Multifocal	47 (20.6%)	23 (17.7%)	24 (25.8%)	0.2
Extra-thyroid extension	3 (1.3%)	1 (0.8%)	2 (2.2%)	0.21
Lympho-vascular invasion	10 (4.5%)	3 (2.3%)	7 (7.5%)	0.065
Perineural invasion	2 (0.9%)	1 (0.8%)	1 (1.1%)	0.32
Lymph nodes	11 (4.9%)	7 (5.4%)	4 (4.3%)	0.71
ATA classification for recurrence risk of differentiated thyroid cancer				
Low	72 (69.9%)	35 (66.0%)	37 (74.0%)	0.378
Intermediate	26 (26.8%)	16 (30.8%)	10 (22.2%)	0.189
High	5 (5.2%)	2 (3.8%)	3 (6.7%)	0.42

Abbreviations: NIFTP: noninvasive follicular-like thyroid neoplasm with papillary-like nuclear features; PTC: papillary thyroid cancer; FTC: follicular thyroid cancer; MTC: medullary thyroid cancer; ATA: American Thyroid Association. * Others: 1 insular, 1 sclerosing, 1 hobnail variant. ^†^ Others: 1 solid variant.

**Table 4:** Predictors of malignancy in indeterminate thyroid nodules (univariate analysis).

	Malignant Frequency (Percentage)	Benign Frequency (Percentage)	*p* Value
* Age (years)	42 (35–51)	43 (36.5–54.5)	0.234
	Sex		
Male	12 (12.6%)	10 (9.5%)	0.48
Female	83 (87.4%)	95 (90.5%)	
Underlying thyroid hormonal status			
Hypothyroidism	4 (4.0%)	5 (4.7%)	0.79
Hyperthyroidism	1 (1.0%)	2 (1.9%)	0.55
Euthyroid	93 (94.9%)	95 (93.2%)	0.60
* TSH level (uIU/mL)	1.3 (0.8–2.3)	1.4 (0.9–2.6)	0.34
Ultrasound features			
Nodule type			
Solid	68 (65.4%)	61 (54.0%)	0.08
Cystic	18 (17.3%)	16 (14.2%)	0.524
Mixed	18 (17.3%)	36 (31.9%)	0.012
Nodule echogenicity			
Hypoechoic	48 (46.2%)	38 (33.6%)	0.059
Hyperechoic	32 (30.8%)	46 (40.7%)	0.12
Isoechoic	24 (23.1%)	29 (25.7%)	0.65
Other high-risk features			
Solid and hypoechoic	34 (32.7%)	20 (17.7%)	0.01
Microcalcifications	39 (37.5%)	18 (15.9%)	0.003
Taller than wide	11 (10.6%)	3 (2.7%)	0.018
Irregular margins	1 (1.0%)	2 (1.8%)	0.58
Extra-thyroid extension	6 (5.8%)	6 (5.3%)	0.9
Lymph nodes	31 (29.8%)	14 (12.4%)	0.002
Ultrasound risk classification systems			
ATA risk classification			
Benign	0 (0%)	1 (0.9%)	0.33
Very low–low suspicion	38 (36.5%)	59 (52.7%)	0.02
Intermediate–high suspicion	66 (63.5%)	53 (47.3%)	0.017
TI-RADS classification			
TI-RADS 1	2 (1.9%)	2 (1.8%)	0.9
TI-RADS 2–3	32 (32.0%)	56 (52.8%)	0.0026
TI-RADS 4–5	68 (68.0%)	50 (47.2%)	0.002

Abbreviations: TSH: thyroid-stimulating hormone; ATA: American Thyroid Association; TI-RADS: Thyroid Imaging Reporting and Data Systems. *: Represented in median (Q1-Q3).

**Table 5:** Predictors of malignancy in indeterminate thyroid nodules (multivariate analysis).

	ORs (95% CI)	*p* Value
Age	1.5 (0.59–3.84)	0.4
Male sex	0.99 (0.97–1.01)	0.38
Hypothyroidism	1.8 (0.11–18.62)	0.799
Hyperthyroidism	1.6 (0.1–24.7)	0.736
TSH level	0.9 (0.8–1.1)	0.28
Ultrasound features		
Nodule Type		
Solid	1.43 (0.8–2.57)	0.23
Cystic	1.49 (0.7–3.17)	0.302
Mixed	0.62 (0.31–1.24)	0.176
Nodule Echogenicity		
Hypoechoic	1.69 (0.98–2.93)	0.06
Hyperechoic	0.55 (0.3–1.02)	0.059
Isoechoic	0.66 (0.33–1.3)	0.228
Other high-risk features		
Solid and hypoechoic	2.26 (1.2–4.26)	0.012
Microcalcifications	3.07 (1.52–6.19)	0.002
Taller-than-wide shape	2.81 (0.7–11.26)	0.144
Irregular margins	0.42 (0.03–5.2)	0.499
Extra-thyroid extension	0.83 (0.23–3.01)	0.776
Lymph nodes	2.43 (1.14–5.18)	0.021
Ultrasound risk classification systems		
ATA risk classification		
Intermediate–high suspicion	1.9 (1.1–3.3)	0.018
TI-RADS classification		
TI-RADS 4–5	2.3 (1.3–4.2)	0.003

Abbreviations: OR: odds ratio; CI: confidence interval; TSH: thyroid-stimulating hormone; ATA: American Thyroid Association; TI-RADS: Thyroid Imaging Reporting and Data Systems.

**Table 6:** Diagnostic performance of ultrasound features and risk stratification systems in the prediction of malignancy.

Ultrasound Feature/Risk Stratification Category	Sensitivity	Specificity	PPV	NPV
Solid and hypoechoic	32.7%	82.3%	63.0%	57.1%
Microcalcifications	37.5%	84.1%	68.4%	59.4%
Lymph nodes	29.8%	87.6%	68.9%	57.6%
ATA intermediate-high suspicion	63.5%	53.1%	55.5%	61.2%
TI-RADS 4–5	68.0%	52.8%	57.6%	63.6%

Abbreviations: PPV: positive predictive value; NPV: negative predictive value; ATA: American Thyroid Association; TI-RADS: Thyroid Imaging Reporting and Data Systems.

## Data Availability

The data presented in this study are available upon request from the corresponding author.

## Abbreviations

The following abbreviations are used in this manuscript:

FNA	Fine-needle aspiration
ATA	American Thyroid Association
KFHU	King Fahad Hospital of the University
KFSH-D	King Fahad Specialist Hospital-Dammam
KFMMC	King Fahad Military Medical Complex
QCH	Qatif Central Hospital
TSH	Thyroid-stimulating hormone
T4	Thyroxine
T3	Triiodothyronine
TI-RADS	Thyroid Imaging Reporting and Data System
Q1 Q3	First quartile Third quartile
OR	Odds ratio
CI	Confidence interval
ETE	Extra-thyroidal extension
PTC	Papillary thyroid carcinoma
FTC	Follicular thyroid carcinoma
PPV	Positive predictive value
NPV	Negative predictive value
